# A Systematic Review of Serum Biomarkers Anti-Cyclic Citrullinated Peptide and Rheumatoid Factor as Tests for Rheumatoid Arthritis

**DOI:** 10.4061/2011/815038

**Published:** 2011-09-11

**Authors:** Peter Taylor, Juliane Gartemann, Jeanie Hsieh, James Creeden

**Affiliations:** ^1^Kennedy Institute of Rheumatology Division, Imperial College, London W6 8LH, UK; ^2^Roche Diagnostics, Ltd., Forrenstra*β*e, 6343 Rotkreuz, Switzerland

## Abstract

This systematic review assesses the current status of anti-cyclic citrullinated peptide (anti-CCP) and rheumatoid factor (RF) tests in the diagnosis and prognosis of rheumatoid arthritis (RA). We reviewed publications on tests and biomarkers for early diagnosis of RA from English-language MEDLINE-indexed journals and non-MEDLINE-indexed sources. 85 publications were identified and reviewed, including 68 studies from MEDLINE and 17 non-MEDLINE sources. Anti-CCP2 assays provide improved sensitivity over anti-CCP assays and RF, but anti-CCP2 and RF assays in combination demonstrate a positive predictive value (PPV) nearing 100%, greater than the PPV of either of the tests alone. The combination also appears to be able to distinguish between patients whose disease course is expected to be more severe and both tests are incorporated in the 2010 ACR Rheumatoid Arthritis Classification Criteria. While the clinical value of anti-CCP tests has been established, differences in cut-off values, sensitivities and specificities exist between first-, second- and third-generation tests and harmonization efforts are under way. Anti-CCP and RF are clinically valuable biomarkers for the diagnosis and prognosis of RA patients. The combination of the two biomarkers in conjunction with other clinical measures is an important tool for the diagnosis and management of RA patients.

## 1. Introduction

Rheumatoid arthritis (RA), the most commonly occurring form of inflammatory polyarthritis, is prevalent in approximately 0.8% of adults worldwide [1]. Approximately 1.3 million adults in the United States have been diagnosed with RA [2]. If untreated, 20%–30% of RA patients become so severely debilitated within the first three years following initial diagnosis that they become permanently disabled [1]. 

Within the last decade, treatment options for RA patients have improved dramatically. Treatment focus has shifted to early intervention with aggressive treatment aimed at suppressing inflammation and preventing further joint damage [3]. Recent evidence indicates that early introduction of methotrexate (MTX) therapy in undifferentiated arthritis (UA) patients seropositive for anti-CCP delays differentiation to RA and retards the progression of joint destruction [4]. However, initiation of treatment without a confirmed diagnosis of RA is inappropriate for at least half of patients with UA [5], as therapies are potentially toxic and costly. 

**Figure 1:**
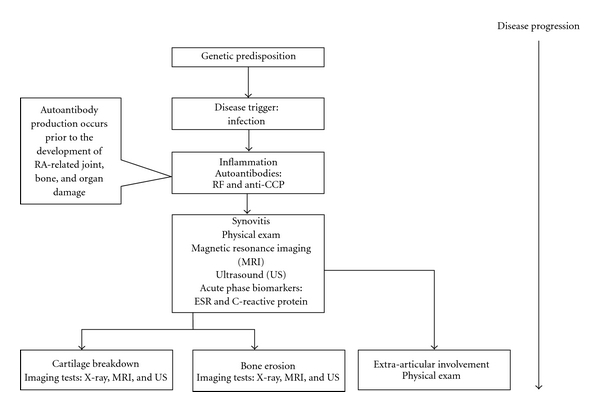
Pathogenesis in rheumatoid arthritis and current diagnostic tools in use.

The shift to earlier, aggressive treatment would be facilitated by more sensitive and specific early diagnostic tests. However, this remains challenging given the limitations of current diagnostic tools and intervention practices [6]. Clinical evidence, laboratory tests, and imaging tests are used to diagnose patients suspected of RA [5, 7, 8]. However, clinical examinations often fail to identify patients with early RA due in part to heterogeneity of disease presentation and course [5]. A systematic review of publications evaluating the performance of the ACR's 1987 clinical criteria found the sensitivity to early RA to be between 77% and 80%, with specificities between 33% and 77% based on pooled data [9].  Figure 1 summarizes the pathogenesis in RA and current diagnostic tools in use.

For patients with a confirmed diagnosis of RA, the course of their disease can vary substantially. In some cases, disease progression can be slow and the overall impact of the disease rather mild, remaining a predominantly local condition. However, for other patients the disease progression can be quite aggressive with significant joint erosion and systemic impact [6, 10]. Accurate prognostic information would be valuable in determining the appropriate treatment course for these different patient populations and improving patient outcomes.

To meet the need for improved diagnostic and prognostic tests and algorithms, various serum biomarkers are being assessed for the improved diagnosis and prognosis of RA, including a wide range of autoantibodies. However, only rheumatoid factor (RF) and anti-cyclic citrullinated peptide (anti-CCP) have gained wide acceptance. We systematically reviewed the literature pertaining to the diagnosis and prognosis of RA to assess the current status of these two autoantibodies as diagnostic and prognostic tests for RA.

## 2. Methods

We reviewed MEDLINE-indexed publications using search strategies that included the keywords “rheumatoid arthritis” and one of the following: (1) diagnos* OR monitor* OR identif* OR detect*, (2) biomarker* OR circulating OR serum, and (3) guideline* OR practice pattern* OR treatment pattern*. We also searched for the keyword “anti-CCP” as a unique search criterion. The limits for these searches included: Field: Title/Abstract, Limits: published between August 2003 and March 2010, only items with abstracts, humans, English, Spanish. A search for relevant guidelines included the following limits: published in the last 10 years, only items with abstracts, humans, English, Spanish. The searches yielded 551 publications. 

**Figure 2:**
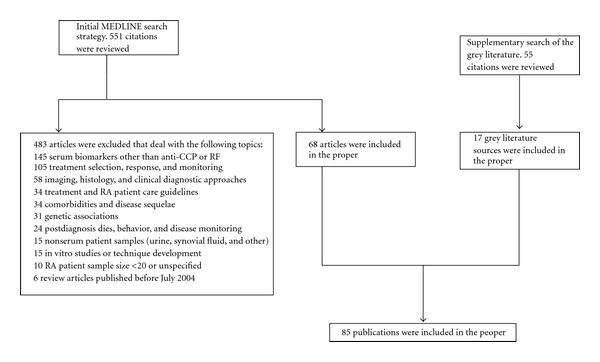
Literature retrieval strategy.

All 551 abstracts were reviewed in depth to identify articles evaluating the characteristics, performance, and clinical utility of anti-CCP and RF tests for use in the diagnosis of RA. Given the broad search criteria applied intended to capture the largest number of relevant publications possible, a total of 483 abstracts were excluded based on the exclusion categories listed in Figure 2. In total, 68 MEDLINE-indexed publications were retrieved in full text.

We also searched the “grey” literature (material that can be referenced, but is not published in peer-reviewed, MEDLINE-indexed medical journals) for information pertaining to anti-CCP and RF for use in the diagnosis of RA. We conducted a search on the Google search engine using the keywords used in MEDLINE searches. Further, a search of guidelines related RA diagnosis was performed on the website maintained by the National Guideline Clearinghouse. The abstracts for the American College of Rheumatology's 7th Annual Conference and the European League Against Rheumatism (EULAR) Annual Congress 2008 also were searched using keywords. Fifty-five publications relevant to the study's aim were selected for further review. 

Primary research articles from the search of MEDLINE-indexed articles that were included in this paper were assessed for the quality of evidence provided using the Oxford ratings system defined by the Center for Evidence-Based Medicine (CEBM). The Oxford ratings define five levels of evidence, with level 1 being the highest level of quality and level 5 the least. Level 1 research studies may be used to support the highest grade A of clinical practice recommendations, level 2 and 3 studies support grade B recommendations, while level 4 and level 5 studies may support grade C and grade D recommendations, respectively [11] (see Table 1).

## 3. Results

We identified 68 articles from MEDLINE pertaining to current diagnostic guidelines and practices for RA, and to anti-CCP and RF as diagnostic tests for RA. Non-MEDLINE sources comprised of current practice guidelines and conference abstracts yielded an additional 17 articles, for a total of 85 publications reviewed for this study (see Figure 2).

Of the MEDLINE publications, 48 were evaluated for their quality of evidence using the Oxford ratings system's diagnostic criteria, one was evaluated using the Oxford ratings system's prognostic criteria, six were evaluated using both the diagnostic and prognostic criteria, and 13 articles were nonsystematic review articles for which the ratings were not applicable. Of the 54 diagnostic publications evaluated, 37 met the requirements for a level 1 quality of evidence that could support grade A recommendations. Three publications met the level 2 requirements and could support grade B recommendations, while the remaining 14 met the level 4 requirements and could support grade C recommendations. Of the prognostic publications, four met the requirements for level 1, two for level 2, and one for level 4. Overall, the diagnostic studies were able to meet a very high level of quality of evidence given the availability of the ACR RA classification criteria as an appropriate reference standard and the high level of specificity of the anti-CCP tests. For the prognostic studies, the requirement for level 1 is a prospective cohort study with good followup, a standard that the majority of the studies were able to meet [11].

### 3.1. Pathology of Citrullinated Proteins and Autoantibodies in RA

Posttranslational modification of the amino acid arginine to the amino acid citrulline is a naturally occurring process mediated by protein-arginyl deaminase (PAD) enzymes [12, 13] Citrullination is currently thought to be involved in cell differentiation and programmed cell death or apoptosis [12]. The naturally occurring targets of PAD identified to date are important structural proteins [12], and citrullination appears to mark these proteins for degradation [12, 14]. Upon cell death, the citrullinated proteins are released into the blood stream [15]. 

Van Venrooij and colleagues have hypothesized that in RA, a triggering event results in the migration of inflammatory cells into the joints and that activates of PAD enzymes, stimulating apoptosis of the joint cells. For the vast majority of individuals, the citrullinated proteins are cleared normally; however, in the 1% of patients with a genetic predisposition, the citrullinated proteins fail to be cleared properly and elicit the production of autoantibodies, which in turn initiate an inflammatory response [15]. A number of species of citrullinated proteins have been identified in inflamed synovial tissue in RA, including vimentin, *α*-enolase and the *α*- and *β*-chains of fibrin [12]. To date, over 100 autoantibody species recognizing citrullinated proteins have been identified in the sera of RA patients [16].

### 3.2. Tests for Anticitrullinated Protein Antigens (ACPAs)

We found 38 primary research studies which evaluated tests for ACPAs. Of these, 18 used a cohort study design that validated ACPA test results within a defined patient population, and 20 were case-control studies comparing ACPA results in RA patients versus control populations. Thirty of these studies met the standards for level 1 evidence, while eight of the case-control studies only met the requirements for level 4 evidence. 

Given the relationship between citrullinated proteins and RA, autoantibodies recognizing citrullinated proteins are attractive biomarkers of RA, and tests measuring these autoantibodies have been in development for the past two decades [17–20]. The first of these tests, referred to as anti-perinuclear factor (APF) and antikeratin antibodies (AKA), was found to measure antibodies in the sera of RA patients which recognized the citrullinated protein filaggrin [17–20]. While not physiologically relevant given that citrullinated filligrin is not produced in synovial joints, these tests proved useful nonetheless in the identification of RA patients [12, 19]. In an effort to improve test performance, Schellekens et al. developed synthetic linear peptides based on filaggrin to be used as the antigen in serum RA tests [17, 21]. 

Antibody recognition was further improved by the development of cyclic peptides rendering the citrulline moiety more available to serum antibodies; these tests were referred to as anti-cyclical citrullinated protein tests or anti-CCP tests [17, 21]. Second- and third-generation anti-CCP tests use combinations of synthetic cyclic citrullinated peptides selected through a screening process to increase sensitivity over anti-CCP1 while continuing to maintain specificity [17, 22].  Table 2 provides a summary of first-, second-, and third- generation anti-CCP tests identified in this literature review.

Recently, Pruijn and colleagues compared published sensitivities of anti-CCP2, anti-CCP3, and anti-MCV tests in comparative studies using the same specificity values for the three biomarkers. The sensitivities of the anti-CCP2 tests were equal to or better than those of the anti-CCP3 and anti-MCV tests across all studies [24, 35, 56, 59–64]. 

A number of tests have been developed that measure the presence of serum antibodies recognizing specific citrullinated proteins for use as biomarkers of RA. In particular, autoantibodies against vimentin (referred to as anti-MCV and anti-SA) and *α*-enolase have attracted interest for applications in RA testing [23, 45, 65–72]. Assays for RA are also being developed recognizing antibodies against modified citrullinated peptides, like chimeric fibrin/filaggrin [73].

### 3.3. Harmonization of Anti-CCP Tests

Given that all three generations of anti-CCP tests as well as other ACPA tests are currently available commercially, there has been a growing recognition that the results of these tests are not consistent across different laboratories and that harmonization of ACPA test results is needed [35, 63, 74]. Several authors have discussed important difficulties in comparing test results across ACPA tests including differences in cutoff values, the need for a common reference material, and the need to include noncitrullinated antigens in ACPA tests to control for nonspecific binding of ACPAs to arginine residues [35, 63, 74–76].  Table 2 underscores the need for harmonization of anti-CCP tests, illustrating the huge variation in cutoff values and performance characteristics of first-, second-, and third- generation anti-CCP tests.

Among current efforts at harmonization of ACPA tests are the development of international reference reagents by the Centers for Disease Control and Prevention [63] and the IUIS Autoantibody Standardization Committee [74], and the establishment of a bank of sera from patients with RA and other rheumatic diseases by the European AutoCure consortium for use in diagnostic test comparisons [63].

### 3.4. Anti-CCP as a Predictive Tool for RA

We identified six primary research articles that assessed anti-CCP tests as predictive tools for RA. Of these, three were cohort studies, two were case-control studies, and one was a systematic review of the anti-CCP literature. Four studies met the criteria for level 1 evidence, one met the level 2 requirements, and one met the criteria for level 4 evidence.

Anti-CCP is present in the serum of a portion of RA patients and has been identified in the serum of patients at all stages of RA: preclinical, early, and established. In clinical research studies, anti-CCP antibodies were found in 55%–69% of patients with RA, [65, 77] 65% of RA patients with late-onset RA [78], and 10% of patients with juvenile-onset arthritis [77]. Blood bank studies have identified anti-CCP antibodies in the serum of patients as many as 12 to 14 years prior to the development of RA [79–82]. In these studies, 34%–40% of the RA patients had anti-CCP+ results prior to disease onset [79–82]. The length of time that anti-CCP antibodies are detectable in patient serum prior to disease onset appears to be age related. Serum anti-CCP is detectable in the serum of older patients well before the developmental of clinical symptoms, while in younger patients, the detection of serum anti-CCP occurs closer to the time of disease onset [83]. 

In patients with UA, anti-CCP+ serum can indicate patient risk of developing RA [27]. One study of UA patients with anti-CCP2+ serum reported an odds ratio (OR) of 25 [12, 84]. The difference between patients who produce anti-CCP antibodies and those who do not may be partly genetic. A study of UA patients who were later diagnosed with RA found an important association between anti-CCP and the shared epitope (SE) genetic predisposition [85]. The presence of anti-CCP has been associated with HLA-DRB1 alleles known to carry SE [17, 86, 87]. SE has also been shown to be associated with the production of antibodies against a range of citrullinated proteins found in inflamed synovial tissue, including vimentin and *α*-enolase [65]. The combination of anti-CCP+ serum and a class II MHC allele with SE appears to be associated with an increased patient risk of developing RA, as evidenced by a reported OR of 66.8 for patients with the combination versus an OR of 25.1 for patients with anti-CCP+ serum only [17, 88].

### 3.5. Anti-CCP as a Diagnostic Tool for RA

We identified 37 primary research studies that evaluated anti-CCP tests of which 18 used a cohort study design that validated anti-CCP tests for use as diagnostic tools within a defined patient population and 19 were case-control studies comparing anti-CCP results in RA patients versus control populations. Twenty-eight of these studies met the standards for level 1 evidence, while eight of the case-control studies could only meet the requirements for level 4 evidence. 

Recent clinical trials suggest that anti-CCP is highly accurate in selectively identifying RA patients [39, 89, 90], and to be of particular diagnostic use in patients who are negative for RF [90]. Current research appears to support the hypothesis that RA patients who are positive or negative for anti-CCP antibodies may constitute two subsets of the RA syndrome with different clinical presentations over time [63]. As shown in Table 2, anti-CCP tests overall have very high specificity but moderate levels of sensitivity; this is in keeping with the hypothesis that different patient populations exist that are either anti-CCP positive or anti-CCP negative (see Table 2). Synovial tissue from anti-CCP+ patients expresses higher concentrations of immune cytokines, has higher numbers of infiltrating lymphocytes, and shows a greater degree of joint destruction than tissue from anti-CCP-negative (anti-CPP−) patients [91]. Even within anti-CCP+ patient populations, subgroups may be differentiated by the quantity of serum antibodies recognizing citrullinated peptides and specific anti-CCP isotypes [67, 92]. 

A meta-analysis of 18 studies of anti-CCP found that the pooled OR for development of RA related to a positive anti-CCP2 test is 16.8, a strong indication of its diagnostic value [93]. However, the absence of anti-CCP is diagnostically less helpful, and in the case of a patient presenting with persistent peripheral joint swelling of four or more weeks duration, a negative test should not be used as a reason not to refer. Indeed, the poor negative predictive value is an argument to discourage antibody testing in the primary care arena.

Until recently, established guidelines did not include anti-CCP as a recommended laboratory test for RA diagnosis [94, 95]. The absence of anti-CCP tests in RA guidelines was largely due to the fact that current-generation anti-CCP tests were developed within the past few years and that their performance characteristics were still being studied in clinical settings. However, in 2007 the European League Against Rheumatism (EULAR) recommended that all patients diagnosed with early arthritis be tested for the presence of anti-CCP [6], and the 2008 RA treatment guideline published by the American College of Rheumatology (ACR) includes positive anti-CCP as one of a number of measures of poor patient prognosis to be used in treatment selection [96]. Over the past three years, the ACR and EULAR jointly developed an updated set of criteria for RA to replace the outdated 1987 ACR classification criteria for RA. The new 2010 Rheumatoid Arthritis Classification Criteria include the presence of anticitrullinated protein antibodies as one of the criteria to be used in determining whether or not a patient should be diagnosed as having RA [97]. The guidelines also mention that ACPAs are tested as anti-CCP [97].  Table 3 provides a summary of the 2010 Rheumatoid Arthritis Classification Criteria.

### 3.6. Anti-CCP as a Differential Diagnostic Tool for RA

We identified fourteen primary research articles that compared anti-CCP test results in RA patients and in patients with diseases that could be confounded with RA. Of these, the vast majority (13 of 14) were case-control studies, while two were cohort studies. Seven studies met the criteria for level 1 evidence, one study met the level 2, criteria and six of the case-control studies met the level 4 criteria.

RA shares symptoms with other forms of arthritis as well as with other disease conditions [1]. Diseases considered as part of the differential diagnosis for RA include other connective tissue diseases including systemic lupus erythematosus and scleroderma, systemic diseases such as infective endocarditis and rheumatic fever, spondyloarthropathies including psoriatic arthritis, infectious arthritis, crystal-induced arthritis or gout, endocrinopathies including thyroid disorders, soft tissue syndromes and degenerative disorders such as fibromyalgia and polyarticular osteoarthritis, deposition disorders such as hemochromatosis, and malignancies such as lung cancer and multiple myeloma [98]. 

Beyond serving as one of several criteria used in the current classification of RA [97], the results of anti-CCP tests also may provide useful clinical information for the differential diagnosis of RA [12]. Numerous studies have compared anti-CCP levels in RA patients with control groups comprised of mixed non-RA rheumatic diseases (osteoarthritis, systemic lupus erythematosus (SLE), and Sjogren syndrome (SS)), spondyloarthropathies (psoriatic arthritis, ankylosing spondylitis), systemic sclerosis, crystal-induced arthritis, gout, infectious arthritis, systemic sclerosis, fibromyalgia, and/or other related conditions as well as healthy controls [23, 24, 27, 42, 44–46, 51, 54, 72]. For specificities in the range of 91.5% to 98.8%, anti-CCP tests have shown sensitivities between 60.2% and 83.3% [23, 24, 27, 44–46, 51, 54, 72]. These results indicate that while not sufficient for a diagnosis of RA as in the case of anti-CCP-negative patients, the presence of elevated anti-CCP levels can help support the differential diagnosis of RA and the above indications.

Two clinical studies comparing anti-CCP levels in the sera of RA patients with levels measured in the sera of small samples of patients with other arthritic conditions have found anti-CCP antibodies to be absent in the sera of patients with osteoarthritis, polymyalgia rheumatica, crystal arthritis, and postinfective polyarthritis [48, 99]. Although anti-CCP antibodies have been measured in the serum of some patients with hepatitis C, SLE, leprosy, juvenile idiopathic arthritis, psoriatic arthritis, and SS, anti-CCP tests have been shown to be able to differentiate RA from these other conditions [15, 85, 99–104]. In patients who developed either RA or other forms of arthritic disease including osteoarthritis, SLE, spondyloarthropathy, neoplastic syndrome, and SS, Kudo-Tanaka and colleagues found that serum anti-CCP levels in the 18 RA patients were consistently >15 U/mL, while that of the 54 patients who developed non-RA arthropathies was <15 U/mL. The only exception was three patients diagnosed with non-RA arthropathies but with pre-RA symptoms whose serum anti-CCP levels were above 15 U/mL [27]. 

Anti-CCP has also been shown to detect early RA in patients with active tuberculosis [105]. For SLE patients with chronic destructive/deforming arthritis and patients with pulmonary tuberculosis who are anti-CCP positive, the ratio of anti-CCP to unmodified arginine-containing peptide (CAP) differs between these and RA patients, providing a means for differential diagnosis [85, 106–108]. Interestingly, in a study of patients with Down's syndrome, over half of the patients were positive for anti-CCP; however, none of the patients had clinical signs of arthritis and there is currently no known association between anti-CCP-positive sera in Down's patients and RA [101].

### 3.7. Anti-CCP as a Prognostic Tool for RA

We identified seven primary research studies evaluating anti-CCP as a prognostic tool for RA. All seven studies used a prospective cohort design. Four studies met the criteria for level 1 evidence, two studies met the level 2 criteria, and one study met the level 4 criteria.

Anti-CCP correlates with various aspects of RA and can be a powerful predictor of disease course. The ESCISIT recommends anti-CCP as one of several prognostic tools for the identification of patients with persistent and/or erosive disease [6]. Several studies have demonstrated a strong association between the presence of anti-CCP and joint damage, with higher titers of anti-CCP antibodies in the serum of patients with erosive RA [10, 47, 48, 50, 77, 109]. One study, however, failed to show a statistically significant relationship between anti-CCP and erosive disease [44]. In a large, prospective study of RA patients followed over 10 years, the presence of anti-CCP antibodies was the most robust single predictor of radiographic progression in patients with early RA. For these patients, even low or moderate levels of anti-CCP antibodies resulted in an OR of 2.6 for the patient developing radiographic progression, while for high levels of anti-CCP antibodies, the OR reached 9.9 [110]. Other studies have shown that anti-CCP is capable of predicting patient development of erosive disease and hand deformity [10, 12, 40, 90, 111, 112]. The presence of anti-CCP at diagnosis is also capable of predicting higher levels of disease activity as measured by the number of swollen joints, DAS28 and other clinical measures [113]. 

Studies have shown anti-CCP in combination with other biomarkers can provide even greater prognostic power. Serum anti-CCP antibodies in patients with the shared epitope is associated with poorer radiological outcome [36]. Risk of severe disease was significantly higher in anti-CCP+ patients who carry one or two HLA-DRB1 alleles with SE [85, 114]. Average expectancy rate for radiological progression was 10 times higher in patients with the combined presence of anti-CCP2 antibodies, RF, and SE, than in patients lacking these three biomarkers [115, 116]. Grade of disease activity was more accurately defined by combination tests for the presence of anti-CCP antibodies and alleles HLA-DR-B1*04 with SE [115].

### 3.8. Rheumatoid Factor

RF, an antibody recognizing the Fc or conserved portion of human antibodies [117], is present in 60%–90% of RA patients with established RA [8, 118] but in less than 50% of patients with early RA [118]. Three-to-five percent of healthy adults have serum RF; this increases to 10%–30% in the elderly [12, 119]. IgA, IgG, and IgM forms of RF have all been identified in RA patients as well as pan-specific and Ga-specific forms of IgM RF [34, 69, 120, 121]. The three isotypes appear at different times pre-clinically, with IgM-RF being present the furthest in advance of diagnosis (3.8 years) followed by IgA-RF (3.2 years) and IgG RF (0.9 years) [122]. 

RF is more established as a biomarker for RA than anti-CCP, having been adopted as one of the ACR's classification criteria for RA in 1987 [123]. The European Standing Committee for International Clinical Studies Including Therapeutics (ESCISIT) notes that it is one of several prognostic markers used to identify patients with persistent and/or erosive disease but does not recommend RF as a diagnostic marker for RA [6] most likely at least in part due to its limited specificity. RF is also common in other autoimmune diseases, infectious diseases, and malignancies, making it a relatively nonspecific marker of RA [3, 8]. Chronically high titers of RF are thought to be more specific for RA [118] as well as being prognostic of poor outcomes [94].

### 3.9. Anti-CCP and RF for Treatment Monitoring of RA

The benefit of anti-CCP tests for treatment monitoring is controversial, with inconsistent findings on the extent and duration of altered anti-CCP antibody levels following RA treatments. In some studies, anti-CCP antibody levels tended to remain stable in RA patients following treatment or decreased only slightly [79, 85, 124]. Other studies observed significant reductions in anti-CCP levels following treatment only in patients whose disease duration was less than one year [125, 126]. Significant reductions in anti-CCP levels have been reported in patients with established RA following treatment with tissue necrosis factor (TNF) blockers and in patient groups with positive clinical responses following treatment with infliximab, etanercept, and adalimumab [85, 124, 125, 127–130]. However, several studies have shown that treatment of established RA patients with TNF-*α* inhibitors reduces RF levels while having little or no effect on anti-CCP levels [17, 131–133]. These conflicting results may be due in part to differences in subjects' disease duration as well as the length of followup [125]. 

Several studies have shown significant differences in treatment effects on serum RF and anti-CCP antibody levels. In studies showing reduced anti-CCP levels following treatment, these reductions were smaller than reductions seen in IgM-RF levels in patients with established RA [125]. RF levels were shown to be significantly reduced by treatment with infliximab with or without MTX [131–133], whereas anti-CCP antibody levels were unaffected by treatment with infliximab [132, 133] and were only temporarily reduced before returning to baseline levels by treatment with both infliximab and MTX [131]. Further, Mikuls et al. found RF levels to decrease progressively in correlation with the clinical course of disease, while anti-CCP levels decrease only partially within the first year of disease and in a manner that correlates neither with treatment nor with clinical course [17, 126].  Table 4 summarizes the findings from these studies.

These distinctions may yield information about differential effects of treatment intervention on short- and long-lived autoantibody-producing plasma cells at different stages of disease evolution or even according to treatment approach, but they are unlikely to add value to standard clinical measures as a means to monitor therapeutic response. However, in recently reported subanalysis of pooled data from two phase III trials of the anti-CD20 biologic rituximab in RA, patients who were seropositive for RF and/or anti-CPP, were two-to-three times more likely to achieve ACR responses to rituximab than those seronegative for both autoantibodies [134], suggesting a potentially important role for both biomarkers in treatment selection. These promising data suggest the potential for personalized medicine approaches using RA biomarkers to determine the most appropriate treatments for RA patients.

### 3.10. Anti-CCP and RF in RA Pathology

Diagnostic and prognostic studies comparing anti-CCP antibodies and RF in the same RA patient samples suggest that the presence or absence of these biomarkers in patient sera may define patient subgroups with potentially different outcomes (see Table 5). Also, RA treatment has significantly different effects on serum RF and anti-CCP antibody levels. These results have led to the hypothesis that RF and anti-CCP are part of two separate antibody systems representing different but overlapping physiological processes, with RF regulated by TNF-*α* and anti-CCP independent of such regulation [17]. 

This hypothesis may be further corroborated by genetic risk factor studies. Several RA susceptibility genes have been identified, including HLA-DRB1 with the shared epitope (SE) and PTPN22 [150, 151]. The combination of either of these susceptibility genes with smoking appears to place patients at significantly higher risk of developing antibodies against citrullinated proteins [150, 152–154]. Interestingly, while HLA-DRB1 SE, PTPN22, and smoking were each independent factors associated with the production of antibodies against citrullinated proteins with an odds ratio of 11.1, only HLA-DRB1 SE and smoking were independent factors associated with the production of RF with a much lower odds ratio of 4.4 [150], supporting the hypothesis that these autoantibodies are produced by different pathological processes.

### 3.11. Anti-CCP and RF Test Performance

We identified eight primary research articles that directly compared the performance of anti-CCP and RF tests, half of which were cohort studies, and the other half were case-control studies. Six of the primary research studies met the criteria for level 1 evidence while two of the case-control studies satisfied the criteria for level 4.

Historically, Anti-CCP has been viewed as highly specific to RA but not as sensitive as RF. Pooled data from a meta-analysis of over 5,000 RA patients give RF a test sensitivity of 62% and a test specificity of 87% [93]. Studies have reported the specificity of anti-CCP to be in the range of 95%-96%, with a sensitivity of 53%–58%. [46, 89, 99]. However, a meta-analysis of 56 studies found the sensitivity of anti-CCP2 to be 68% with a specificity of 95%, suggesting that it may in fact be more sensitive than RF [85, 90]. A similar analysis of 29 RF studies showed RF only to have a sensitivity of 60% with a specificity of 79% [155, 156]. 

The absolute values of the two biomarkers' sensitivities and specificities range considerably across the studies identified by this paper. This is due, in part, to studies using different cutoff values for anti-CCP and RF, and different test methodologies and manufacturers. In two studies, however, cutoff values corresponding to a predefined test specificity of >98.5% were applied to serum tests of both biomarkers, allowing for a head-to-head comparison of sensitivities for anti-CCP and RF. The resulting sensitivities were 73.7%–77.7% for anti-CCP but only 7.4%–12.8% for RF [36, 111]. In a more recent study, Wild et al. found similar results when the cutoff value was set at 95% specificity, with anti-CCP identifying 77% of RA patients while RF identified only 62% [157]. Thus, anti-CCP appears to have better sensitivity and specificity than RF. A meta-analysis also showed anti-CCP to have higher odds ratios for the prediction of developing RA and for radiographic progression than RF, indicating that anti-CCP may be the better prognostic tool [93]. 

However, this does not mean that anti-CCP can replace RF in diagnostic and prognostic testing for RA. Of RF-patients, approximately 20% are anti-CCP+ [17, 158–161]. Of anti-CCP-patients, a comparable 15%–20% are RF-positive [17, 162–164]. Approximately 30% of RA patients are reported to be negative for both anti-CCP and RF [136]. The two tests therefore appear to be complementary, with anti-CCP of particular diagnostic value for RF-patients [79, 109]. In combination, the two tests appear to be even more powerful [109] with a positive predictive value (PPV) nearing 100%, greater than the PPV of either of the tests alone [17, 99, 165]. The presence of both RF and anti-CCP antibodies has also been shown to predict which patients will develop RA [17, 166, 167]. Interestingly, Shovman et al. found the highest correlation of serum RF and anti-CCP in nonerosive patients; however, the reason for this correlation is not clear [50]. Improved diagnostic results for the combination of anti-CCP and RF have been shown when RF is measured using a multiplex cytofluorimetric assay [168]. Table 5 summarizes characteristics of patients positive and negative for anti-CCP and RF.

### 3.12. Prediction Algorithms for RA in Development

Given the limitations of individual tests to predict RA development and disease progression, predictive algorithms are being developed that combine relevant patient demographics with various test measures, including combinations of serum biomarkers. Biomarker combinations that show promise as future diagnostic or prognostic panels for early RA include anti-CCP, anti-MCV, and IgM-RF [66], anti-CCP and matrix metalloproteinases 3 (MMP-3) [169], and anti-CCP, MMP-3 and high-sensitivity CRP (hsCRP) [170]. Elevated anti-CCP, MMP-3 and hsCRP were all seen preclinically [170]. The combination of anti-CCP, RF and CRP was observed to predict development of RA [31]; however, another study showed anti-CCP by itself to be superior in predicting RA development over any combinations with RF, CRP, MMP-3, and antigalactosyl IgG antibody (CARF) [27]. The combination of anti-CCP or other anticitrullinated protein antibodies with RF and sE-selectin also has shown both diagnostic and prognostic potential [171].

## 4. Discussion

RF tests are well established as diagnostic and prognostic tools for RA and have been incorporated as a criterion in the ACR's classification of RA patients since 1987 [123]. The recent addition of ACPA testing in the ACR's updated 2010 RA classification criteria is an acknowledgment of the clinical value of these biomarkers for the diagnosis of RA patients [97]. Alone, RF and anti-CCP tests are not sufficient for the diagnosis of RA, since not all RA patients have both biomarkers and some patients lack both (see Table 5). However, in combination with other clinical measures, the two biomarkers together provide important diagnostic and prognostic information about different RA patient populations depending on which of the biomarkers if any are present in patient sera (see Tables 3 and 5). 

Anti-CCP tests have evolved significantly over the past 20 years. From the initial linear antigens used to identify serum autoantibodies against citrullinated proteins, the development and refinement of cyclic citrullinated antigens has led to improvements in test sensitivity while maintaining extremely high levels of specificity [17, 22]. Anti-CCP tests have been shown to be both more sensitive and more specific a test for RA than RF [36, 85, 90, 93, 111, 155, 156]. This paper identifies a large number of diagnostic studies providing level 1 evidence of the clinical value of anti-CCP tests in the diagnosis of RA supporting the validity of anti-CCP tests for this application. Fewer studies were reviewed that evaluated the prognostic value of anti-CCP tests and these provided mixed levels of evidence. Given the promising results to date that anti-CCP is indicative of more severe disease courses, it would be beneficial for additional prospective, longitudinal studies with good patient followup to be conducted to further validate the prognostic value of anti-CCP tests.

Three generations of anti-CCP tests are currently available commercially. Of the three generations, the second generation tests currently appear to provide the best performance [24, 35, 56, 59–64]. Given different cutoff levels, sensitivities and specificities, there has been a growing recognition that anti-CCP results are not interchangeable across laboratories [35, 63, 74]. The ongoing development of international reference standards is a critical element in efforts to harmonize anti-CCP test results in order to make the interpretation of these results consistent and improve the ability of clinicians to make patient management decisions [63, 74]. 

In conclusion, Anti-CCP and RF are recognized biomarkers associated with RA that are incorporated in the current ACR classification guidance for RA diagnosis. The studies reviewed indicate that the two biomarkers are promising early diagnostic tests with the potential to support early, aggressive intervention using newer RA treatment options (see Figure 1). Anti-CCP appears to be a strong predictor of erosive RA, making it a potentially important prognostic tool that could be used to inform patient management decisions. Additional prospective longitudinal research studies of the prognostic value of anti-CCP tests would help to validate this clinical application. Finally, the adoption of international reference standards will significantly improve the consistency of anti-CCP results across laboratories.

**Learning Points**

- The preclinical presence of anti-CCP and RF makes these important biomarkers for early RA.
- Newer-generation anti-CCP assays provide higher sensitivity and specificity than RF.
- At comparable specificity, the sensitivities of anti-CCP2 tests are superior to those of anti-CCP1 tests and equal to or better than those of anti-CCP3 tests.
- Anti-CCP and RF levels may reflect different pathological pathways in RA and, in combination, can identify the majority of patients with RA.
- The absence of anti-CCP is diagnostically less helpful in a patient presenting with persistent peripheral joint swelling.
- *∼*50% of new-onset RA patients (fulfilling ACR criteria by 18/12 follow up) are anti-CCP positive [84].
- Early treatment of *anti-CCP positive* UA (not fulfilling ACR criteria for RA) with MTX slows development of both full-blown RA and joint damage (PROMPT STUDY) [4].
- Recent findings suggest that patients positive for anti-CCP and/or RF will respond better to anti-CD20 therapeutics than patients seronegative for both autoantibodies.
- Anti-CCP positivity is an important predictor of radiographic progression in RA patients.

##  Key Messages

- Anti-CCP preclinical presence makes it an important biomarker for early RA.
- Newer generation anti-CCP assays offer high specificity, and combined with RF tests, greater sensitivity.

##  Funding

This work was supported by Roche Diagnostics, Ltd. The statements contained in this paper are solely those of the authors, and no endorsement by Roche Diagnostics, Ltd. should be inferred or implied.

##  Conflict of Interests

J. Creeden, J. Gartemann, and J. Hsieh are employees of Roche Diagnostics.

## Figures and Tables

**Table 1:** Oxford Centre for Evidence-based Medicine definitions of levels of evidence. The following table summarizes the criteria that must be satisfied for the five different levels of evidence for both diagnostic and prognostic studies as well as the grading of recommendations assessment, development, and evaluation (GRADE) that studies with a given level of evidence will support.

Level of evidence	Grade of recommendation studies can support	Criteria for diagnostic studies	Criteria for prognostic studies
1	A	(a) systematic review/meta analysis of level 1 studies or clinical decision rule applied to level (b) multicenter studies (b) cohort study with good reference standards that validate the diagnostic test or clinical decision rule in single center study (c) specificity or sensitivity results that are so high that they can rule in or rule out a diagnosis	(a) systematic review/meta analysis of inception cohort studies or clinical decision rule that is validated in more than one population (b) single inception cohort studies with ≥80% follow up of participants or clinical decision rule that is validated in one population only (c) case series where results apply to either all or none of the subjects

2	B	(a) systematic review/meta analysis of ≥level 2 diagnostic studies (b) exploratory cohort study with good reference standard or clinical decision rule applied to a split sample	(a) systematic review/meta analysis of retrospective cohort studies or untreated control groups in randomized clinical trials (RCTs) (b) retrospective cohort study or untreated control group from an RCT that is followed over time (c) outcomes research

3	B	(a) systematic review/meta analysis of ≥level 3 diagnostic studies (b) nonconsecutive study or study with inconsistent use of reference standards	N/A

4	C	Case-control study or study with poor reference standards	Case series or poor quality cohort studies

5	D	Expert opinion	Expert opinion

(http://www.cebm.net/index.aspx?o=1025).

Source: GRADE (grading of recommendations assessment, development, and evaluation) Working Group 2007 1 (modified by the EBM Guidelines Editorial Team).

**Table 2:** Anti-CCP tests.

Manufacturer/test type	Test*	Cut point (U/mL)	Sensitivity (%)	Specificity (%)	References
Abbott Laboratories	Anti-CCP	5	69.4	97.6	[23]
	AxSYM	5	80.6	84.3	[24]
	Anti-CCP2				
Axis-Shield Diagnostics	Anti-CCP	7.2	52.8	100	[25]
	Anti-CCP2	4.5	82.4	82.8	[26]
		4.6	83.3	93	[27]
		5	53.7–67.5	90.4–99.3	[28–31]
		5.6	88	81	[32]
		25	71	65	[10]
Eurodiagnostica	Anti-CCP	20.7	52.8	100	[25]
		53	47	94	[33]
		65	43.8	98.4	[34]
	Anti-CCP2	5	66.7	97	[29]
		25	66.7–76.5	92.2–97.3	[29, 35–37]
		42	73.7	98.6	[36]
Euroimmun	Anti-CCP	5	39.2–65	95.2–99	[38–41]
		14.8	81.6	87.5	[42]
		25	56	96	[43]
	Anti-CCP2	5	66.7–72.5	96.4–97.7	[29, 35]
		5.25	78.7	95.6	[44]
Fuchun-Zhongnan Biotech	Anti-CCP2	25	61.8	96.3	[45]
Genesis	Anti-CCP2	6.25	69.6	93.9	[35]
Immunoscan	Anti-CCP2	25	54–64.4	96–97.1	[46, 47]
In-house ELISA test	Anti-CCP1	92	42	97	[47]
INOVA Diagnostics	Anti-CCP	6.4	58.5	100	[25]
	Anti-CCP2	20	60.2–78.6	70.3–98.8	[22, 24, 29, 35, 48–52]
		25	82	96	[53]
		30	70	91.5	[54]
	Anti-CCP3	20	61.3–82.9	93–97.6	[24, 29, 49, 51]
MBL Co.	Anti-CCP	3.8	72.8	92	[55]
Phadia GmbH	Anti-CCP2	7	77.5	95.9	[35]
		10	70.1	98.7	[56]
Pharmacia Diagnostica	Anti-CCP2	7	80.3	97	[22]
Roche Diagnostics	Anti-CCP2	9	69	93	[57]
		13.6	92.2	86.2	[58]
		17	90.6	86.8	[58]

*The generation of anti-CCP test indicated is as reported in the methods section of the articles referenced.

**Table 3:** 2010 ACR/EULAR scoring criteria for RA. The following table provides the scoring criteria for different domains of evaluation. For each cell in the table for which the patient satisfies the condition, the cell is scored by the number of points at the top of the column in which it is found. The patient's score is the sum of the scores for the individual cells. Patients with a total score of 6 or more points are diagnosed as having RA.

Number of points	0	1	2	3	5
Joint involvement	1 medium-large joint	2–10 medium-large joints	1–3 small joints	4–10 small joints	>10 small joints

Serology	Negative for both RF and anti-CCP	Positive for either RF or anti-CCP at low titer (between 1*x* and 3*x* upper limit of normal)	Positive for either RF or anti-CCP at high titer (>3*x* upper limit of normal)		

Duration of synovitis	<6 weeks	≥6 weeks			

Acute phase reactants	normal CRP and ESR	abnormal CRP or ESR			

Source: [97].

**Table 4:** Studies of anti-CCP and RF levels following treatment for RA.

Study	Subjects	Serum Tests	Results
Alessandri et al., 2004 [124]	Prospective cohort study of 43 patients with RA not responding to DMARDs treated with infliximab in combination with methotrexate	Serum samples collected and tested for anti-CCP antibodies and RF at baseline and after 24 weeks	(i) Serum titres of anti-CCP and RF decreased significantly after 24 weeks of treatment (anti-CCP −14%; RF −20%) (ii) Significant decreases in serum anti-CCP antibodies and RF observed only in patients with clinical improvement

Atzeni et al., 2006[128]	57 patients with RA not responsive to methotrexate treated with adalimumab as part of the ReAct open-label phase IIIb study	Serum samples collected and tested for anti-CCP antibodies and RF at baseline and after 24 and 48 weeks of followup	(i) Treatment resulted in significant decreases in anti-CCP serum levels at 24 weeks (−14%) and 48 weeks (−33%) (ii) Treatment resulted in significant decreases in RF serum levels at 24 weeks (−33%) and 48 weeks (−42%) (iii) The decrease in anti-CCP and RF antibody titers correlated with the clinical response to the therapy

Bobbio-Pallavicini et al., 2004 [131]	Prospective study of 30 consecutive patients with RA; patients were followed during 78 weeks of infliximab and methotrexate therapy for refractory rheumatoid arthritis	Serum samples collected and tested for anti-CCP antibodies and RF at baseline and after 30, 54 and 78 weeks	(i) % patients positive for RF, Anti-CCP approximately same at baseline and 78 weeks (ii) Median RF titre underwent progressive reduction from 128 IU/mL to 53 IU/mL (iii) Anti-CCP antibody titre significantly decreased at 30 weeks but returned to baselinev

Caramaschi et al., 2005 [133]	Prospective cohort study of 27 patients with refractory RA treated with infliximab and methotrexate	Serum samples collected and tested for anti-CCP antibodies, Rf and CRP at baseline and after 22 weeks	(i) Serum levels of anti-CCP antibodies did not change from baseline with infliximab treatment (ii) IgM RF and CRP levels decreased significantly with infliximab treatment

Chen et al., 2006 [130]	90 patients with RA who failed treatment with DMARDs; randomized clinical protocol in which all 90 patients continued DMARD treatment and 52 patients were assigned for additional treatment with etanercept	Serum samples collected and tested for anti-CCP and RFat baseline and one month intervals for three months during the treatment course	(i) Serum anti-CCP levels decreased 31.3% in patients positive for anti-CCP at baseline treated with etanercept (ii) Serum RF levels decreased 36% in patients positive for RF at baseline treated with etanercept (iii) Decreases in serum anti-CCP and RF levels were progressive throughout the three-month treatment course (iv) Changes in anti-CCP levels was positively correlated with changes in various clinical measures of RA

De Rycke et al., 2005 [132]	Prospective cohort study of 62 patients with refractory RA treated with infliximab combined with methotrexate	Serum samples collected and tested for anti-CCP antibodies, IgM RF, CRP and ESR at baseline and after 30 weeks	(i) RF titres significantly reduced at baseline and week 30 during infliximab treatment (ii) Anti-CCP antibodies unchanged by infliximab treatment (iii) IgM RF titres correlated inversely with changes in CRP and ESR; Anti-CCP antibodies did not correlate inversely with these biomarkers

Mikuls et al., 2004 [126]	Retrospective study of serum samples from 66 RA patients who completed double-blind, randomized clinical protocols (1) methotrexate, hydroxychloroquine, and sulfasalazine, (2) minocycline versus placebo, and (3) minocycline versus hydroxychloroquine	Serum samples collected at baseline and at a followup averaging 13.7 months ± 8.6 months; Samples were stored at −80° and later and tested for anti-CCP antibodies and RF	(i) 52% of patients positive for anti-CCP antibodies at baseline had >25% reduction in anti-CCP antibody levels during treatment course (ii) 55% of patients positive for RF at baseline had >25% reduction in RF levels during treatment course (iii) Significant reductions in anti-CCP levels was only seen in patients with disease duration <12 months (iv) No association was seen between reductions in anti-CCP levels and treatment response (v) Significant reductions in RF levels were determined by treatment response

**Table 5:** Characteristics of patients positive and negative for anti-CCP and rheumatoid factor.

Anti-CCP−/RF+	Anti-CCP+/RF+
(i) 5% [135] to ~12%–20% [17, 136–139] of RA patients (ii) 81% of RF+ patients [136] (iii) Intermediate form of RA [135]	(i) 50% [136] to 63% [135] of RA patients (ii) The probability of RA is ~90%–100% [8, 17, 140] (iii) High probability of developing erosive RA [17, 140] (iv) Most severe form of RA [135]

Anti-CCP−/RF−	Anti-CCP+/RF−

(i) 14% [135] to *∼*30% of RA patients [136] (ii) A low probability of RA, but the disease cannot be ruled out [8] (iii) Mildest form of RA [135]	(i) 8% [136] to 18% [135] of RA patients (ii) *∼*20% [17, 141–145] to 40% RF-negative RA patients [12, 146, 147] (iii) Intermediate form of RA [135] (iv) Substantial risk of developing RA [63, 148, 149]
