
JOURNAL INFORMATION
==============================
NLM Title Abbreviation: Autoimmune Dis
Journal ID: Autoimmune Dis
NLM Journal ID: 101546750
Journal ID: AD

Autoimmune Diseases

ISSN: 2090-0422
EISSN: 2090-0430
Publisher: Wiley

ARTICLE INFORMATION
==============================
PMCID: PMC3270500
DOI: 10.1155/2012/734069
Article version: 1
Subjects: Erratum

Erratum to: A Systematic Review of Serum Biomarkers Anti-Cyclic Citrullinated Peptide and Rheumatoid Factor as Tests for Rheumatoid Arthritis

Taylor Peter C. 1
Gartemann Juliane 2
Hsieh Jeanie 2
Creeden James 2 *
1 Kennedy Institute of Rheumatology, Nuffield Department of Orthopaedics, Rheumatology and Musculoskeletal Sciences, University of Oxford Botnar Research Centre, Windmill Road Headington, Oxford OX3 7LD, UK
2 Roche Diagnostics, Ltd., Forrenstraße, 6343 Rotkreuz, Switzerland
*James Creeden: james.creeden@roche.com
Print publication date: 2012
Electronic publication date: 2012 Jan 19
Volume: 2012
Electronic Location ID: 734069
Received 2011 Nov 6; Accepted 2011 Nov 20
Copyright: Copyright © 2012 Peter C. Taylor et al.
Copyright year: 2012
License: This is an open access article distributed under the Creative Commons Attribution License, which permits unrestricted use, distribution, and reproduction in any medium, provided the original work is properly cited.
License URL: https://creativecommons.org/licenses/by/3.0/

Erratum for: A Systematic Review of Serum Biomarkers Anti-Cyclic Citrullinated Peptide and Rheumatoid Factor as Tests for Rheumatoid Arthritis 2011 11 9 2011 815038 Autoimmune Diseases PMC3170888 21915375

==============================
Table 1 2010 ACR/EULAR scoring criteria for RA. The following table provides the scoring criteria for different domains of evaluation. For each cell in the table for which the patient satisfies the condition, the cell is scored by the number of points at the top of the column in which it is found. The patient's score is the sum of the scores for the individual cells. Patients with a total score of 6 or more points are diagnosed as having RA.

Number of points	0	1	2	3	5	
Joint involvement	1 medium-large joint	2–10 medium-large joints	1–3 small joints	4–10 small joints	>10 small joints	
	
Serology	Negative for both RF and anti-CCP		Positive for either RF or anti-CCP at low titer (between 1x–3x upper limit of normal)	Positive for either RF or anti-CCP at high titer (>3x upper limit of normal)		
	
Duration of synovitis	<6 weeks	≥6 weeks				
	
Acute phase reactants	Normal CRP and ESR	Abnormal CRP or ESR				
Source: [145]

